# Exosomes-derived miR-154-5p attenuates esophageal squamous cell carcinoma progression and angiogenesis by targeting kinesin family member 14

**DOI:** 10.1080/21655979.2022.2037322

**Published:** 2022-02-14

**Authors:** Yuwei Shou, Xiaoqian Wang, Yinghao Liang, Xiaonan Liu, Kuisheng Chen

**Affiliations:** a Academy of Medical Sciences, Zhengzhou University, Zhengzhou Henan, China; b Department of Pathology, The First Affiliated Hospital of Zhengzhou University, Zhengzhou Henan, China; c Henan Key Laboratory of Tumor Pathology, Zhengzhou University, Zhengzhou Henan, China

**Keywords:** Exosomes, miR-154-5p, kinesin family member 14 (KIF14), esophageal squamous cell carcinoma (ESCC), angiogenesis

## Abstract

Exosomes participate in the progression and angiogenesis of esophageal squamous cell carcinoma (ESCC). This study aimed to explore the effect and mechanism of exosomes-derived miR-154-5p on the progression and angiogenesis of ESCC. The exosomes with the diameter of 40–270 nm were successfully isolated from ESCC cells by ultracentrifugation. They were then assessed by transmission electron microscope (TEM), nanoparticle tracking analysis (NTA), and Western blotting. Kinesin family member 14 (KIF14) was upregulated, while miR-154-5p was downregulated in ESCC as examined by Quantitative Real-time PCR (qRT-PCR). Exosomes-derived miR-154-5p from ESCC cells was found to attenuate the cellular migration, invasion, and angiogenesis of ESCC using Cell Counting Kit-8 (CCK-8), wound healing assay, transwell migration assay, and tumor formation assays. Moreover, KIF14 was proven to be a direct downstream target gene of miR-154-5p in ESCC cells using luciferase assay. In conclusion, our study identified that exosomes-derived miR-154-5p attenuates ESCC progression and angiogenesis by targeting KIF14 *in vitro*, which might provide a novel approach for the diagnosis and treatment of ESCC.

## Graphical Abstract

**Figure:**
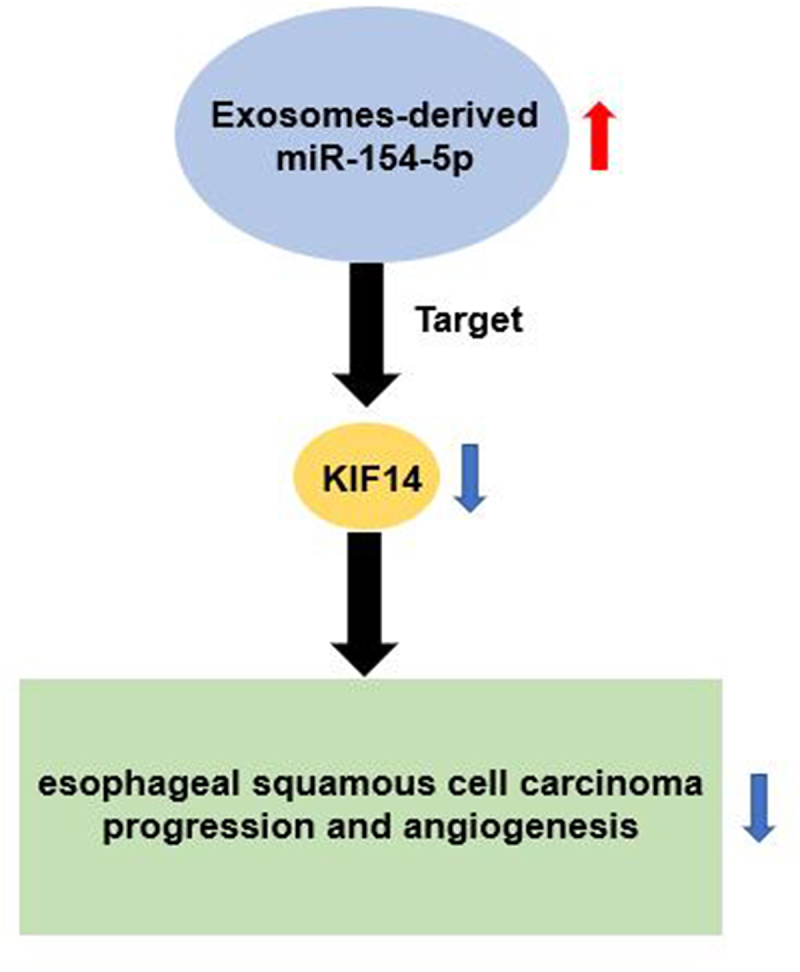

## Introduction

Esophageal cancer ranking as the seventh most prevalent cancer resulted in over 500,000 deaths and 572,000 new cases worldwide in 2018 [1]. Esophageal squamous cell carcinoma (ESCC) is the dominant subtype of esophageal cancer, accounting for 90% of all esophageal cancer cases [2]. Despite the advanced clinical diagnosis and treatment, the 5-year overall survival of ESCC patients is less than 20% due to the rapid progression, recurrence, and metastasis [3]. Angiogenesis is a key indicator for tumor growth and metastasis in ESCC [4–6]. Therefore, investigating the underlying mechanism of angiogenesis in ESCC may provide a novel therapeutic approach for ESCC.

Exosomes, nano-sized bio-vesicles with a diameter of 40–100 nm, are secreted by nearly all mammalian cells after the fusion of multivesicular bodies with the plasma membrane [7]. Exosomes can transport important biological molecules including mRNAs, microRNAs (miRNAs), lipids, and proteins [8]. Exosomes-derived miRNAs participate in ESCC progression, which may be potential diagnostic markers for ESCC [9–11]. MiR-154-5p was found to be a tumor suppressor in multiple cancers such as nasopharyngeal carcinoma [12], breast cancer [13], and colorectal cancer [14]. However, the function of the exosomes-derived miR-154-5p in ESCC progression has not been explored yet.

Kinesin family member 14 (KIF14) belongs to kinesin-3 superfamily that is an essential microtubule-dependent molecular motor [15,16]. Several studies reveal that KIF14 is associated with multiple biological processes such as cytokinesis, cell proliferation, and apoptosis [17–19]. KIF14 was identified as an oncogene in retinoblastoma [20], ovarian cancer [21], and colorectal cancer [19]. In ESCC, KIF14 is regulated by LETM1 and it was also proven to be an oncogene that regulates cellular proliferation, invasion, migration, and angiogenesis [22]. However, the upstream miRNA regulators of KIF14 have not been explored in ESCC.

Our present study aimed to explore the function of exosomes-derived miR-154-5p in ESCC and reveal its regulatory mechanism on KIF14 to regulate ESCC progression and angiogenesis. Our results might offer a novel approach for ESCC diagnosis and treatment.

## Material and methods

### Bioinformatics analysis

GSE38129 from GEO DataSets is a profile having the differentially expressed genes (DEGs) in ESCC samples with reference to non-tumor samples. After setting the criteria of adjusted *p*-value (adj. *p*) < 0.05 and logFC > 2, the upregulated DEGs in ESCC samples were screened. Then, STRING, an online tool to perform gene ontology (GO) enrichment for the upregulated DEGs, was used. Another profile GSE114110 storing the DEGs in ESCC samples compared with non-tumor samples was also downloaded from GEO DataSets. After setting the criteria of adj. *p*< 0.05 and logFC < −2, the downregulated miRNAs were screened. In addition, TargetScan was used to predict the miRNAs putatively targeting KIF14 mRNA.

### Clinical samples and cell lines

The tumorous and adjacent normal tissues were collected from 28 ESCC patients in our hospital between March 2020 and March 2021. All participants signed the informed written consent form before this work was conducted. This work was approved by the ethics committee of our hospital (approval number: 2021-KY-0396-007). The clinical characteristics of all the ESCC patients are listed in Table 1.

**Table 1.** The clinical characteristics of 28 patients with esophageal squamous cell carcinoma

Variable	N (%)
Age (years)	
>60	15(53.6)
≤60	13(46.4)
Sex	
Male	12(42.9)
Female	16(57.1)
KPS	
>80	23(82.1)
≤80	5(17.9)
Pathological T stage	
T1	7(25.0)
T2	12(42.9)
T3	9(32.1)
Lymph node metastasis	
N1	8(28.6)
N2	10(35.7)
N3	10(35.7)
Histology	
Well differentiated	6(21.4)
Moderately differentiated	14(50.0)
Poorly differentiated	8(28.6)
Tumor location	
Upper	10(35.7)
Middle	13(46.4)
Lower	5(17.9)

KPS, Karnofsky Performance Status

All cell lines, including ESCC cell lines (KYSE410 and KYSE150), human umbilical vein endothelial cells (HUVECs), and human esophageal epithelial cell line (HET-1A), were obtained from ATCC (USA). The ESCC cell lines were cultured in RPMI-1640 medium, whereas HET-1A and HUVECs cell lines were cultured in the DMEM medium. Cells were cultured using 10% FBS in a CO_2_ incubator (5% CO_2_) at 37°C.

### Cell transfection

The miR-154-5p mimic (Cat#: miR10000452-1-5), mimic negative control (mimic-NC, Cat#: miR1N0000001-1-5), miR-154-5p inhibitor (Cat#: miR20000452-1-5), and inhibitor negative control (inhibitor-NC, Cat#: miR2N0000001-1-5) were obtained from RiboBio (China). According to the supplier’s protocol, Lipofectamine 2000 (Invitrogen, USA) was used to transfect 50 nM of miR-154-5p mimic, mimic-NC, miR-154-5p inhibitor, and inhibitor-NC into 3 × 10^5^ ESCC cells under the logarithmic growth phase. After 48 h of transfection, the transfection efficiency of miR-154-5p mimic was assessed using qRT-PCR.

### Quantitative Real-time PCR (qRT-PCR)

The extraction of total RNA from tissues and cell lines was conducted by TRIzol reagent (Invitrogen, USA). TransScript One-Step gDNA Removal and cDNA Synthesis SuperMix (Transgene, China) was used to perform the cDNA synthesis. qRT-PCR was performed using SYBR Premix Ex Taq (Takara, China). The relative expression was analyzed using the 2^−ΔΔCt^ method [23]. GAPDH and U6 were used as housekeeping genes for mRNA and miRNA, respectively. The primers’ sequences are listed in Table 2.

**Table 2.** Primer sequences used for RT-qPCR analysis

Gene names	Primer sequences
miR-125b-2-3p	Forward: 5’-CGCGTCACAAGTCAGGCTCT-3’
	Reverse: 5’-AGTGCAGGGTCCGAGGTATT-3’
miR-154-5p	Forward: 5’-GCCTTCGCTCAACTGAATTG-3’
	Reverse: 5’-CTCAACTGGTGTCGTGGAGTC-3’
KIF14	Forward: 5’-CCGACATTACAGATGCACCA-3’
	Reverse: 5’-CTTCATTCCTAAGCCTACACC-3’
U6	Forward: 5’-CTCGCTTCGGCAGCACATA-3’
	Reverse: 5’-CGCTTCACGAATTTGCGTG-3’
GAPDH	Forward: 5’-TGCACCACCACCTGCTTAGC-3’
	Reverse: 5’-GGCATGGACTGTGGTCATGAG-3’

## Exosome isolation

The isolation of exosomes from ESCC cells was performed by differential centrifugation (300 × g, 10 min; 1200 × g, 20 min; and 10,000 × g, 30 min) at 4°C and ultracentrifugation (200,000 × g, 2 h; 100,000 × g, 2 h) at 4°C as performed in a previous study [24]. The exosomes were suspended using 100 μL PBS and stored at −80°C for later use. The exosomes were observed and photographed using a transmission electron microscope (TEM) at 50,000× magnification, and the size of the exosomes was analyzed by nanoparticle tracking analysis (NTA).

## The assessment of cellular viability

The change in cellular viability was detected using the Cell Counting Kit-8 (CCK-8) kit (Beyotime, China) as reported in a previous study [25]. The ESCC cells with a density of 5 × 10^3^ cells/well were incubated in 96-well plates overnight. After the cell transfection or co-culturing with exosomes for 0, 24, 48, and 72 h, 10 μL of CCK-8 reagent was added per seeded well of the 96-well plate and was left to incubate for 2 h. Finally, the OD was measured at 450 nm using a micro-reader (Bio-Rad, USA).

## The detection of cell migration

After transfection or co-culturing with exosomes, the ESCC cells were seeded into 6-well plates and were left to incubate until they reached more than 90% confluence. Afterward, a 200 μL pipette tip was used to make artificial scratches. Cells were washed with PBS to remove the detached cells and were left to incubate for 24 h. Finally, the wound closures were observed at 0 and 24 h using a light microscope and images were taken as described in a previous study [26].

## The detection of cell invasion

After transfection or co-culturing with exosomes, ESCC cells were plated in the upper chamber of transwell coated with Matrigel and free-medium. The medium with 10% FBS was added to the bottom chamber. After 14 hours, the cells in the bottom chamber were fixed using 4% paraformaldehyde and dye using 0.1%. The images on invaded cells were photographed using a light microscope as described in a previous study [25].

## Western blotting

Total protein was extracted from ESCC cells using RIPA lysis buffer (Beyotime, China) and was then separated by 10% sodium dodecyl sulfate-PAGE. The separated proteins were transferred into PVDF membranes. After blocking the membranes with skim milk for 1 h, the membranes were incubated with CD63 antibody (Cat#: ab271286, Abcam, USA), CD81 antibody (Cat#: ab109201, Abcam, USA), KIF14 antibody (Cat#: ab71155, Abcam, USA), and GAPDH antibody (Cat#: ab9485, Abcam, USA) overnight at 4°C. The following day, the membranes were incubated with anti-HRP-rabbit antibody at room temperature for 3 h. Subsequently, a SuperEnhanced chemiluminescence detection reagent (Applygen, China) was added to the membranes and was left to incubate for 15 min after covering with a plastic wrap. Finally, the blot was visualized using an X-ray film.

### In vitro *angiogenesis*

Tube formation assay was used to assess the *in vitro* angiogenesis as described in a previous study [4]. Fifty microliters of thawed Matrigel matrix was added to each well of 96-well plate and was left to incubate at 37°C for 30 min. The HUVEC cells were then seeded into the 96-well plate with a seeding density of 1000 cells/well. After 8 h, images of tube formation were taken under a light microscope. The number of blood vessel forming nodes was calculated to assess angiogenesis.

## Luciferase assay

The potential binding site of KIF14 3ʹUTR (AUAACCUG) to miR-154-5p was retrieved from TargetScan. The pGL3 vector (Promega, USA) was used to prepare the wild-type (WT) constructs, with the binding sites of KIF14 3ʹUTR, and the mutant (MUT) constructs, without the binding sites of KIF14 3ʹUTR. Then, the WT or MUT KIF14 constructs were co-transfected into KYSE410 and KYSE150 cells together with miR-154-5p mimic or mimic-NC. After 48 h, luciferase activity was detected by Dual-Luciferase Reporter Assay System (Cat#: E1910, Promega, USA).

## Statistical analysis

Paired Student’s test and one-way or two-way analysis of variance were used to analyze the data using GraphPad Prism (version 7). The data were shown as mean ± standard deviation from at least three independent experiments. Results having *p* values < 0.05 were considered statistically significant.

## Results

In this study, bioinformatics analysis identified that miR-154-5p and KIF14 were key regulators in ESCC. Therefore, our study aimed to explore the effect and mechanism of exosomes-derived miR-154-5p on the progression and angiogenesis of ESCC. Using a series of cell functional experiments, our data showed that exosomes-derived miR-154-5p could inhibit the progression and angiogenesis of ESCC through directly targeting KIF14, which might be a novel approach for the diagnosis and treatment of ESCC.

## miR-154-5p and KIF14 might be key regulatory factors in ESCC

**Figure 1.**
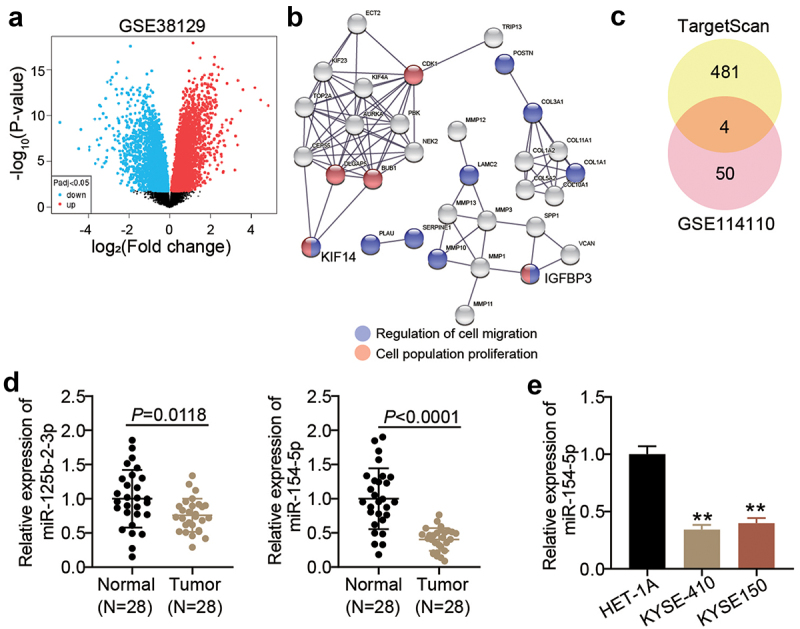
miR-154-5p and KIF14 might be key regulatory factors in ESCC. (a) The DEGs in ESCC samples from GSE38129 were screened with adj. *p* < 0.05 and logFC > 2. (b) GO enrichment for 64 upregulated DEGs was analyzed by STRING. (c) Four common miRNAs (miR-378a-5p, miR-125b-2-3p, miR-154-5p, and miR-338-3p) were overlapped from TargetScan and GSE114110. (d) The expression of miR-125b-2-3p and miR-154-5p in ESCC tissues and adjacent normal tissues was measured by qRT-PCR. (e) The miR-154-5p expression in ESCC cell lines (KYSE410 and KYSE150) and human esophageal epithelial cell line (HET-1A) was measured by qRT-PCR. **p < 0.001 vs HET-1A.

The GSE38129 profile from GEO DataSets stored the DEGs in ESCC samples compared with non-tumor samples (Figure 1a). After setting the criteria (adj. *p* < 0.05 and logFC > 2), 64 upregulated DEGs in the ESCC samples were retrieved. The GO enrichment results from STRING showed that KIF14 and IGFBP3 participate in the cellular migration and proliferation (Figure 1b). Since the regulatory function of IGFBP-3 in ESCC had been previously reported [27,28,29], KIF14 was identified as our target gene of interest.

TargetScan predicted 485 miRNAs that could potentially target KIF14. Moreover, after setting the criteria (adj. *p* < 0.05 and logFC < −2) for the GSE114110 profile storing the mRNA expression data in ESCC samples and non-tumor samples, 54 downregulated miRNAs in ESCC samples were retrieved. Only four miRNAs, namely miR-378a-5p, miR-125b-2-3p, miR-154-5p, and miR-338-3p, were common between the retrieved results of the GSE114110 profile and TargetScan (Figure 1c). Since the effects of miR-378a-5p and miR-338-3p on ESCC had been previously investigated, miR-125b-2-3p and miR-154-5p were of particular interest in this study. After examining the expression of miR-125b-2-3p and miR-154-5p in the collected samples, it was found that the expression levels of miR-154-5p were lower than those of miR-125b-2-3p in the tumor samples (Figure 1d). Therefore, miR-154-5p was identified as our target miRNA. Moreover, miR-154-5p expression was found to be lower in ESCC cells compared to human esophageal epithelial cell line (Figure 1e).

## miR-154-5p overexpression attenuated the malignancy of ESCC cells

**Figure 2.**
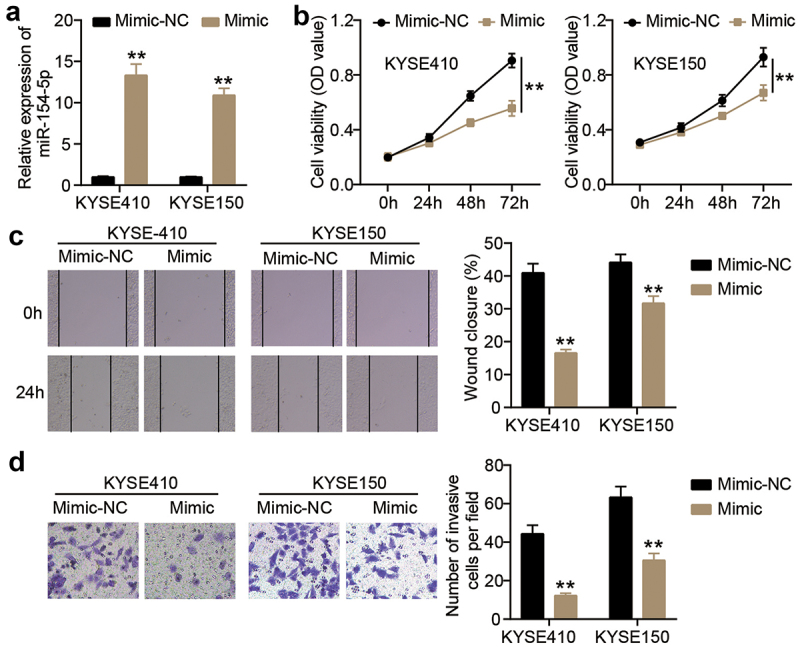
miR-154-5p overexpression inhibited cell malignancy in ESCC cells. (a) The transfection efficiency of miR-154-5p mimic was assessed in KYSE410 and KYSE150 by qRT-PCR. (b) The change of cell viability in ESCC cells after transfecting miR-154-5p mimic was detected by CCK8 assay. (c) The change of cell migration in ESCC cells after transfecting miR-154-5p mimic was detected by wound healing assay. (d) The change of cell invasion in ESCC cells after transfecting miR-154-5p mimic was detected by transwell assay. **p < 0.001 vs mimic-NC. NC, negative control. Mimic, miR-154-5p mimic.

To verify the effect of miR-154-5p on ESCC cells, miR-154-5p mimic was transfected into KYSE410 and KYSE150 cells (Figure 2a). The cellular viability of KYSE410 and KYSE150 cells decreased in miR-154-5p mimic group compared to mimic-NC group (Figure 2b). In addition, the KYSE410 and KYSE150 cells with miR-154-5p mimic showed a significant impediment of cellular migration compared to the cells with mimic-NC (*p* < 0.001, Figure 2c). Moreover, the number of invasive KYSE410 and KYSE150 cells was markedly reduced after transfecting miR-154-5p mimic compared to mimic-NC (Figure 2d).

## miR-154-5p knockdown accelerated the malignant behavior of ESCC cells

After successfully transfecting miR-154-5p inhibitor into KYSE410 and KYSE150 cells (Supplementary Figure 1A), there was an increase in the cellular viability (Supplementary Figure 1B) and aggravation of cellular migration and invasion (Supplementary Figure 1C and 1D, respectively) compared to inhibitor NC.

## Exosomes-derived miR-154-5p attenuates ESCC progression

**Figure 3.**
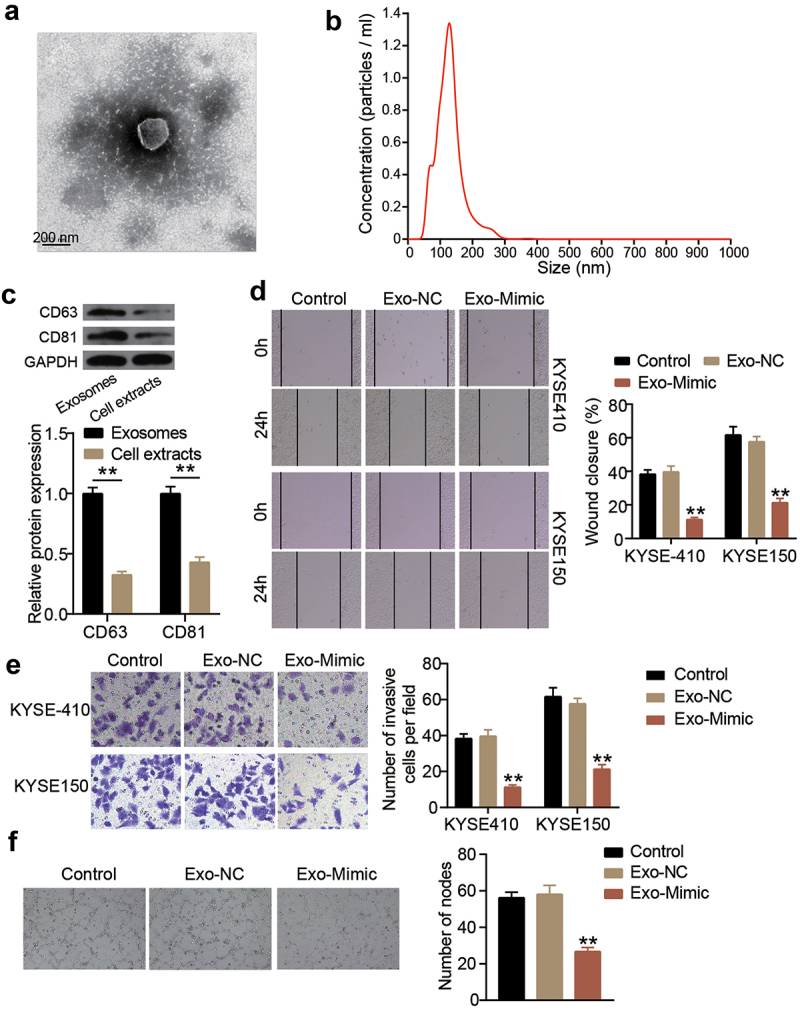
Exosomes-derived miR-154-5p attenuates ESCC progression. (a) The morphological observation of exosomes under TEM at 50,000 × magnification. (b) Nanoparticle tracking analysis of exosomes. (c) The protein expression of exosome markers (CD63 and CD81) was measured by Western blotting. **p < 0.001. (d) The change of cell migration in co-culture of exosomes and ESCC cells was detected by wound healing assay. (e) The change of cell invasion in co-culture of exosomes and ESCC cells was detected by transwell assay. (f) The change of angiogenesis in co-culture of exosomes and ESCC cells was detected by tube formation assay. (d–f) **p < 0.001 vs control. Control, blank control. Exo, exosomes. NC, negative control. Mimic, miR-154-5p mimic.

To verify the function of exosomes-derived miR-154-5p in ESCC cells, exosomes were first isolated. As shown in Figure 3a, upon visualization under a TEM, the exosomes had a spherical-shaped appearance. The NTA showed that the diameter of the isolated exosomes ranged from 40 to 270 nm (Figure 3b). By detecting the levels of the exosome markers, CD63 and CD81, using Western blot, it was found that the expression levels of both CD63 and CD81 were significantly high in exosomes, suggesting the successful isolation of exosomes (Figure 3c). Mimics of miR-154-5p were transfected into the isolated exosomes to upregulate miR-154-5p expression in KYSE410 and KYSE150 cells (Supplementary Figure S2). Afterward, wound healing assay was performed to assess the change in the cellular migration capacity of both ESCC cell lines. The cellular migration capability in the exosomes-derived miR-154-5p group was impaired both in KYSE410 and KYSE150 cell lines (Figure 3d). Furthermore, the transwell migration assay showed that the exosomes-derived miR-154-5p was able to reduce the number of invasive cells (Figure 3e). In addition, the *in vitro* angiogenesis capability was inhibited as observed in the inhibition of tube formation in HUVECs cells upon miR-154-5p overexpression in exosomes (Figure 3f).

## miR-154-5p targeted KIF14 in exosomes from ESCC cells

**Figure 4.**
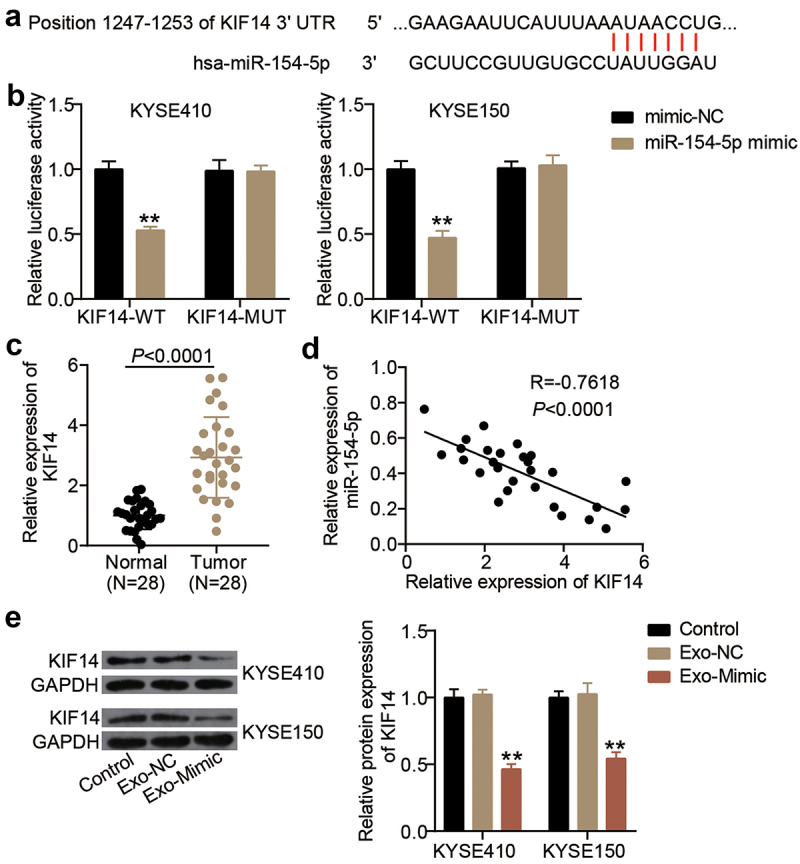
miR-154-5p targeted KIF14 in exosomes from ESCC cells. (a) The binding sites on KIF14 3ʹUTR for miR-154-5p were predicted by TargetScan. (b) The targeting relationship between KIF14 and miR-154-5p was proved by luciferase assay. **p < 0.001 vs mimic-NC. (c) The KIF14 expression in ESCC tissues and adjacent normal tissues was measured by qRT-PCR. (d) The relationship between KIF14 expression and miR-154-5p expression in ESCC tissues was analyzed by Pearson’s analysis. (e) The expression of KIF14 protein in co-culture of exosomes and ESCC cells was detected by Western blotting. **p < 0.001 vs control. Control, blank control. Exo, exosomes. NC, negative control. Mimic, miR-154-5p mimic.

TargetScan predicted a putative binding site for miR-154-5p on the 3ʹUTR of KIF14 transcript (Figure 4a). A luciferase reporter assay was performed using the designed KIF14-WT and KIF14-MUT vectors. The luciferase activity was reduced by miR-154-5p mimic in KIF14-WT vector-transfected cells, whereas the luciferase activity in KIF14-MUT vector-transfected cells did not show a significant effect (Figure 4b). Moreover, the KIF14 mRNA expression showed a threefold upregulation in the ESCC tissues compared to the adjacent normal tissues (Figure 4c). Additionally, KIF14 expression levels were negatively correlated with the expression levels of miR-154-5p in ESCC tissues (Figure 4d). Finally, KIF14 protein levels were significantly suppressed in the exosome-derived miR-154-5p-mimic-transfected cells compared to the control cells (Figure 4e).

## Discussion

Angiogenesis is crucial for ESCC cancer development and progression [30]. This study showed that miR-154-5p is a tumor suppressor in ESCC. Moreover, our results revealed that the exosomes-derived miR-154-5p negatively regulates cellular migration, invasion, and angiogenesis in ESCC cells. In addition, we also found that the exosomes-derived miR-154-5p reduced KIF14 expression in ESCC cells through direct targeting KIF14 3ʹUTR.

MiRNAs, a class of small non-coding RNAs, are identified as key regulators of cancer development through binding to their target genes [31,32]. MiR-154-5p is an anti-tumor miRNA in multiple cancers. For instance, miR-154-5p sponged by lncRNA SNHG5 inhibits proliferation and colony formation of breast cancer cells [13]. Another study found that miR-154-5p overexpression attenuated invasion, migration, and tumor metastasis in nasopharyngeal carcinoma [12]. However, the role of miR-154-5p in ESCC has not been uncovered yet. In our study, we revealed that miR-154-5p attenuated the cellular viability, migration, and invasion of ESCC cells.

Recently, biological research has been directed toward studying the effect of exosomes on cancer progression. Many studies have reported that exosomes are able to regulate malignant progression in many cancers like breast cancer [33], colon cancer [34], and glioblastoma [35]. Zhang et al. [25] reported that the exosomes-derived lncRNA FAM225A promoted ESCC progression and angiogenesis through targeting miR-206, thus promoting NETO2 and FOXP1 expression. Another study showed that the exosomes-derived miR-21 from ESCC cells enhanced angiogenesis and proliferation through targeting SPRY1 [11]. Consistent with previous ESCC studies, we isolated exosomes from ESCC cells by ultracentrifugation. TEM, NTA, and Western blotting proved the successful isolation of exosomes with the diameter ranging from 40 to 270 nm. Our study revealed that the exosomes-derived miR-154-5p is a tumor suppressor miRNA as it suppresses the cellular migration, invasion, and angiogenesis of ESCC, which was different from previous studies.

Accumulating evidence indicates that KIF14 participates in the progression of multiple human cancers [19–21]. For instance, KIF14 was found to be upregulated in colorectal cancer, and its overexpression accelerated colorectal tumorigenesis [19]. In ESCC, only one study revealed that KIF14, which is positively regulated by LETM1, induced cellular proliferation, invasion, migration, and angiogenesis [22]. This study showed that miR-154-5p directly targets KIF14, resulting in decreasing its expression levels in ESCC. Our study is consistent with a previous study by Chen et al. [12] which showed that miR-154-5p targets the oncogene KIF14 in nasopharyngeal carcinoma. Thus, we concluded that the exosomes-derived miR-154-5p could inhibit ESCC tumor progression and angiogenesis through targeting KIF14. Moreover, we showed that the oncogene KIF14 was upregulated in ESCC cells.

Our study uncovered the role and mechanism of the exosomes-derived miR-154-5p in ESCC cells. One of the limitations of this study is that it only explored the effects of the exosomes-derived miR-154-5p in ESCC cells *in vitro*. Further investigations should be carried out to unravel whether the exosomes-derived miR-154-5p could regulate ESCC progression *in vivo*. In addition, the upstream key regulators of the exosomes-derived miR-154-5p need to be explored.

## Conclusion

In conclusion, our study showed that the exosomes-derived miR-154-5p inhibits ESCC progression and angiogenesis through targeting KIF14. Thus, the exosomes-derived miR-154-5p may be a potential diagnostic and therapeutic target for ESCC.

## Supplementary Material

Supplemental Material

## Data Availability

The datasets used and/or analyzed during the current study are available from the corresponding author on reasonable request.
