# Supplementary figures and images for: Exosomes-derived miR-154-5p attenuates esophageal squamous cell carcinoma progression and angiogenesis by targeting kinesin family member 14
Source: Bioengineered. 2022 Feb 14;13(2):4610–20. doi: 10.1080/21655979.2022.2037322 (PMC8973613; doi:10.1080/21655979.2022.2037322)

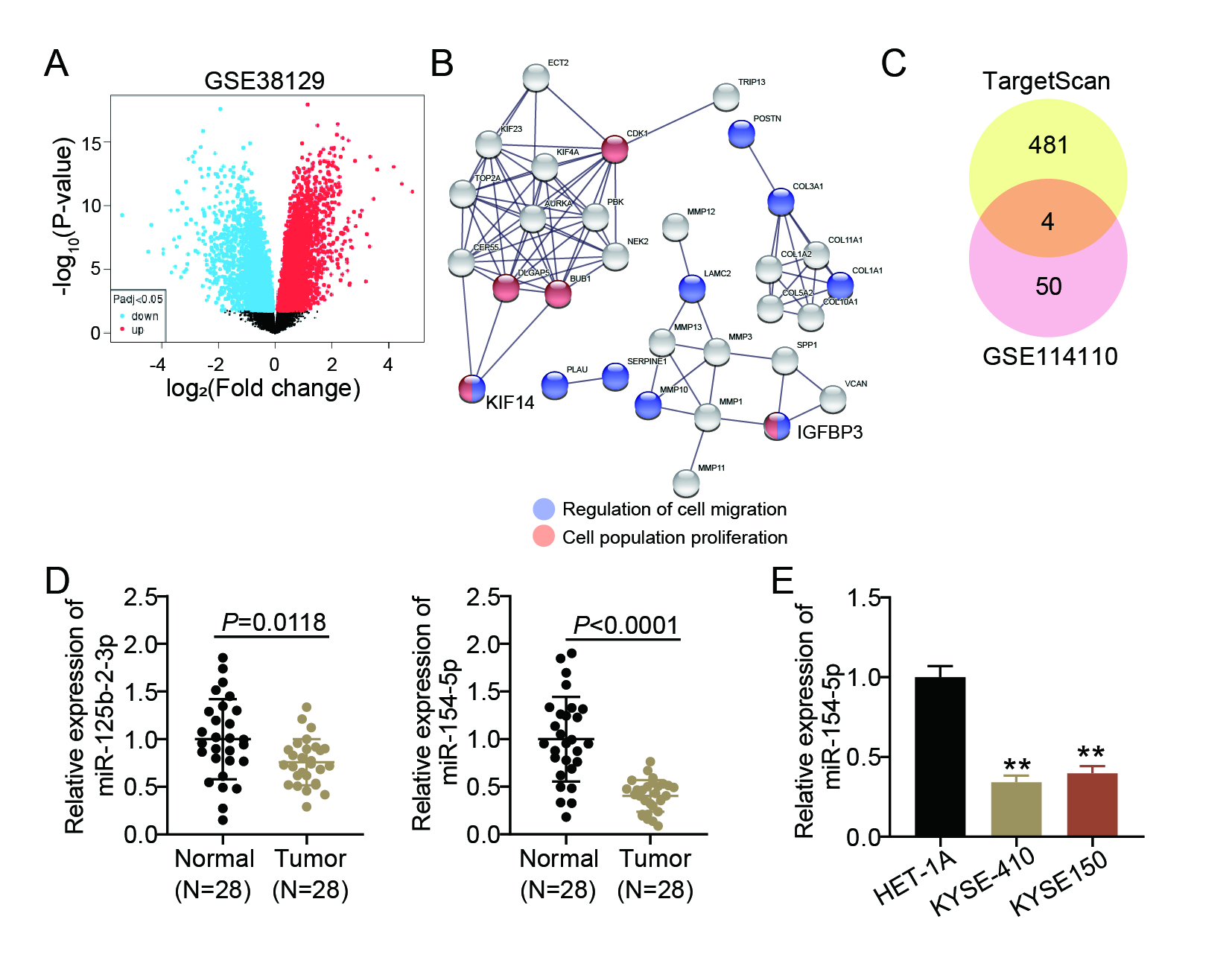

Supplement: Supplemental Material [file KBIE_A_2037322_SM0353.zip › supplementary/Supplementary Figure 1.jpg]

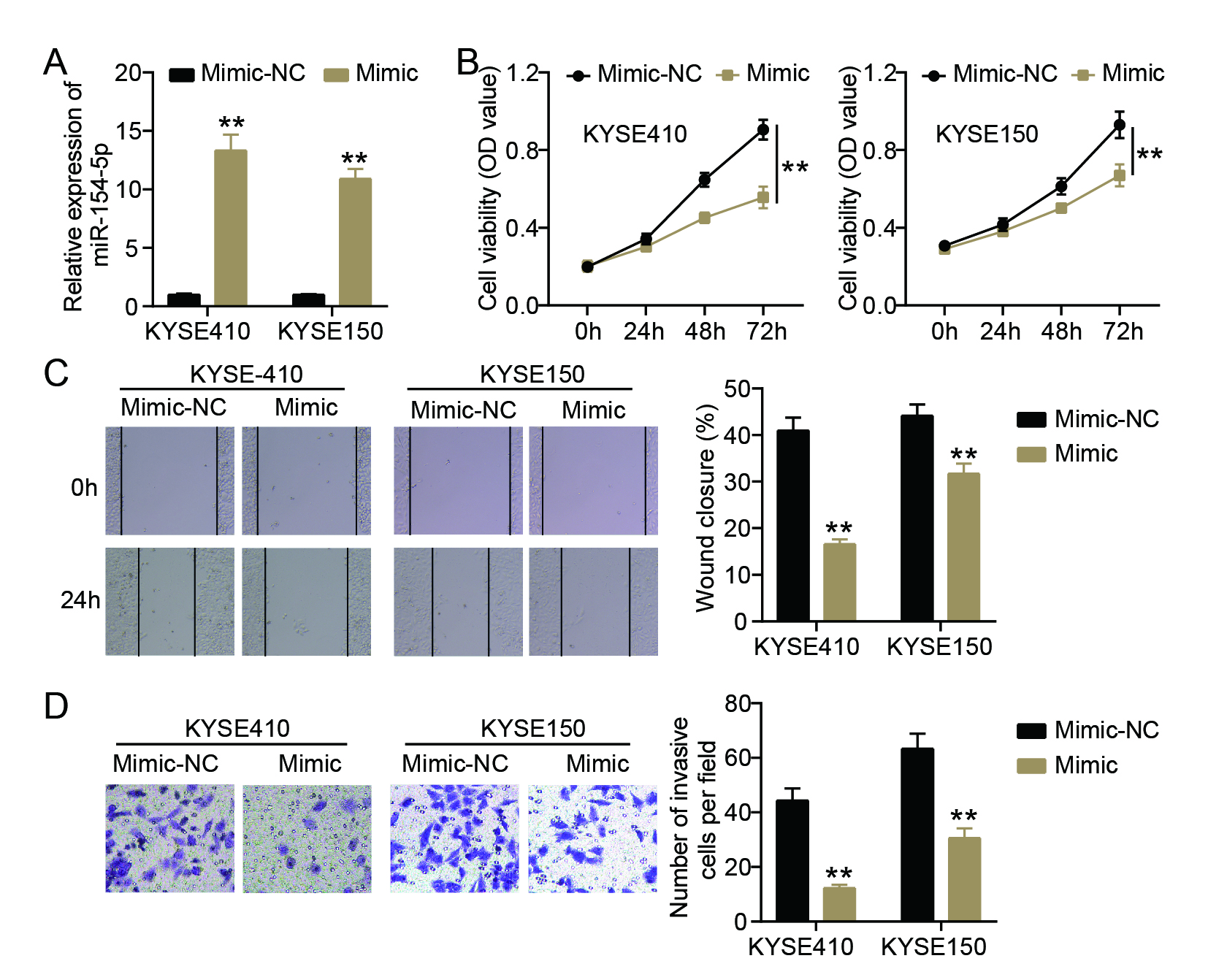

Supplement: Supplemental Material [file KBIE_A_2037322_SM0353.zip › supplementary/Supplementary Figure 2.jpg]
